# Ovarian tissue cryopreservation: An update

**DOI:** 10.4103/0974-1208.44111

**Published:** 2008

**Authors:** Ethiraj B. Prasath

**Affiliations:** KKIVF, Department of Reproductive Medicine, KK Women's and Children's Hospital, 100, Bukit Timah Road, Singapore, 229 899

**Keywords:** Cryoprotectants, freezing, thawing, ovarian tissue cryopreservation, transplantation

## Abstract

Ovarian tissue cryopreservation and transplantation have been considered as promising means of fertility preservation for women who have survived cancer, with livebirths being reported from this technique. Ovarian tissue cryopreservation can be offered to patients with different types of cancer. Among the cryoprotectants, glycerol appears to give the poorest results. The techniques of cryopreserving ovarian tissue and alternative approaches have been reviewed in this article. The readers are reminded that this technique is still experimental and informed consent to be obtained from patients after counseling with medical information on the risks involved.

## INTRODUCTION

Advances in chemotherapy and radiotherapy have increased the survival rate of cancer patients, amazingly up to 90% for young cancer patients. Studies in the US have shown that by 2010, one in 200 individuals will be a survivor of childhood cancer.[1] Increased life span actually raises concerns on the quality of life after treatment for cancer.[2] Having children may be difficult for cancer survivors as both radio- and chemotherapies have been proven to be gonadotoxic.

Recovery of ovarian function after anticancer treatment is very much affected by the loss of follicles due to chemo- or radiotherapy,[3] resulting in premature ovarian failure (POF) and consequently, infertility in many female cancer survivors. Fertility preservation before treatment for cancer is an important option nowadays to overcome infertility induced by cancer itself or by the treatment to cure cancer.

Fertility of female cancer patients could be preserved by various means. The ovaries may be transposed under peritoneum (oophoropexy) to protect them from pelvic irradiation.[4] However, this approach is not suitable for patients undergoing chemotherapy. Assisted reproduction (AR) is another approach to preserve fertility wherein patients may have to undergo controlled ovarian stimulation. Collected oocytes could be fertilized with their partner's sperm through IVF or ICSI and the resulting embryos could be frozen. The patients may use these embryos after their cancer is cured. However, there are some issues in this approach. First of all, cancer patients may not have much time between diagnosis and treatment and AR is time-consuming. Secondly, prepubertal girls and patients without partners are generally not allowed to undergo AR. Thirdly, ovarian stimulation using gonadotropins may not be suitable for estrogen-sensitive cancers. In such situations, oocyte freezing before the treatment for cancer could be offered as an option. Oocyte cryopreservation has yielded successful livebirths in some centers. Nonetheless, it is still experimental and the technique has not been perfected yet. Recently, in ESHRE 2008, vitrification of oocytes has been shown to be promising with a > 90% survival rate. However, slow freezing of oocytes appears to be a golden standard in many centers with varying survival rates. Oocytes may be obtained after simulation with gonadotropins (in nonestrogen-sensitive caners). Otherwise, immature oocytes could be collected and subjected to *in vitro* maturation (IVM) followed by cryopreservation. Alternatively, oocytes could be isolated and cryopreserved from ovarian tissue biopsy or the whole ovary. If the patient had a partner, these oocytes could be fertilized after IVM and the resulting embryos could be frozen.[5,6] However, chances of pregnancy are limited by the number of oocytes or embryos stored. Also, ethical and legal issues may pose some problems. Furthermore, numerous primordial follicles in the ovary would go wasted if oocyte or embryo cryopreservation alone is done.

The ovary has hundreds of primordial follicles containing immature oocytes which are smaller, dormant, less differentiated and without zona.[7] Such immature oocytes could tolerate cryopreservation due to the absence of zona and cortical granules.[8] Ovarian cortex can be obtained from any female cancer patient irrespective of age and marital status with no procedural delays in cancer treatment. Amorim *et al*.[9] have shown that steroidogenic and gametogenic functions are well preserved in cryopreserved ovarian tissue. All these advantages make ovarian tissue cryopreservation a better option than other means for female fertility preservation.

## Ovarian tissue cryoprservation in animals

Ovarian tissue cryopreservation has been practised since the early 1950s.[7] Parrot[10] showed that restoration of fertility is possible in mice after orthotopic grafting of frozen-thawed ovarian tissue. Gosden *et al*.[11] reported the livebirths of young animals after autografting frozen-thawed ovarian tissue in sheep. Similar reports have been published in rats[12] and rabbits.[13] These successful attempts in animals prove that ovarian tissue cryopreservation is a feasible means of fertility preservation with a potential application for human beings.

## Ovarian tissue cryopreservation in humans

Roughly three decades after Parrot's study,[10] reports on successful cryopreservation of human ovarian tissue were published simultaneously by two different groups. Hovatta *et al*.[14] showed that cryopreservation of human ovarian tissue is feasible. They cryopreserved ovarian tissue from 19 patients aged 19–44 years using two different cryoprotectant protocols. Histological investigations of fresh and frozen-thawed ovarian tissues showed similar follicular atresia rates but the morphology of the follicles and oocytes in nonfrozen and frozen-thawed ovarian tissues was not different. The maximum period of tissue storage was five weeks.

Newton *et al*.[15] obtained ovarian cortex from eight donors aged 17–31 years and froze the cortical slices with four different cryoprotectants for two months after which the tissues were grafted into immunodeficient mice. Histological assessment at 18 days after grafting showed the survival of 44–84% of the follicles, paving the way for grafting of cryopreserved ovarian tissue.

Hovatta *et al*.[16] further showed that follicles in *in vitro* cultures of cryopreserved ovarian tissue were viable up to 10–15 days. *In vivo* restoration of ovarian function after cryopreservation and autologous transplantation of ovarian cortex in human beings was first documented by Oktay and Karilkaya.[17] This orthotopic transplantation resulted in follicular development in ovarian tissue pieces grafted beneath the left peritoneum laparascopically in response to menopausal gonadotropin stimulation. Subsequently, Oktay *et al*.[18] reported heterotopic transplantation of the ovarian cortex to the forearm of two patients resulting in restoration of normal endocrine function and follicular growth. In one of the patients, percutaneous oocyte retrieval, after stimulation with gonadotropins, yielded three oocytes of which two were postmature and one was at metaphase I. Although the latter oocyte matured *in vitro* and ICSI was performed, it did not fertilize. Later in 2004, normal embryonic development was reported by the same group in oocytes retrieved from frozen-thawed ovarian cortical strips transplanted beneath the abdominal skin of a breast cancer patient.[19]

The first livebirth from frozen-thawed ovarian cortex after autologous orthotopic transplantation was reported by a Belgian group led by Donnez.[20] Ovarian cortex of a patient with Hodgkin's lymphoma was cryopreserved before cancer therapy. The frozen-thawed cortical strips were transplanted into the peritoneal window beneath the hilum of the inactive right ovary. This resulted in a spontaneous pregnancy and the livebirth of a girl, Tamara. Suspicion on the source of pregnancy was raised by Oktay and Tilly as to whether it arose from transplanted tissue or from the inactive ovary, which could have resumed its function.[21] Donnez *et al*.[20] had replied to this suspicion that they provided endocrinological, laparoscopic and echographic evidence to show that the pregnancy resulted from a follicle grown at the transplantation site. Another spontaneous pregnancy was reported from orthotopic transplantation of frozen-thawed ovarian tissue.[22] Pregnancy from *in vitro* fertilization in a modified natural cycle after the transplantation of cryopreserved ovarian tissue also resulted in livebirth.[23] These livebirths have boosted confidence among oncologists and gynecologists to offer ovarian tissue cryopreservation as a promising means of fertility preservation in women who are threatened by premature ovarian failure due to medical intervention for any cause.

## Indications and age limit for ovarian tissue cryopreservation

Potential indications for ovarian tissue cryopreservation have been listed by various authors.[24–26] Patients diagnosed with breast cancer, cervical cancer, leukemia, lymphoma, Hodgkin's disease, sarcoma, ovarian and endometrial carcinoma, autoimmune diseases, neuroblastoma and other benign and malignant diseases, should be offered ovarian tissue cryopreservation before radio- or chemotherapy. Patients undergoing bone marrow or stem cell transplantation are potential candidates for ovarian tissue cryopreservation because of the high doses of radio- or chemo therapy carried out before transplantation which results in ovarian failure.[27] Ovarian tissue cryopreservation could also be offered to patients suffering from noncancerous disorders such as Turner's syndrome.[25,28] However, ovarian tissue cryopreservation should not be offered for autotransplantation where ovarian involvement in cancer has been confirmed.[29] Therefore, it is mandatory to screen for metastasis into the ovary before offering ovarian tissue cryopreservation. Ovarian metastasis should be ruled out particularly in breast cancer patients as their BRCA gene mutation might result in co-existing ovarian cancer[30] and there is an increased risk of ovarian metastasis in breast cancer.[31] Normal conception and livebirth are possible from transplantation of fresh ovarian cortical strips from one woman to another, if they are monozygotic twins and discordant for premature ovarian failure.[32,33] Putting it simply, ovarian tissue cryopreservation could be offered whenever fertility of female patients has to be preserved or restored, except for ovarian cancer.

At our center, we approached fertility preservation for a married, ovarian cancer patient, in a different way. She underwent oophorectomy after which the ovary was sent to the IVF lab. Six immature oocytes were collected from visible follicles and subjected to *in vitro* maturation with recombinant FSH and LH. ICSI was performed on three mature oocytes using her husband's sperm while two were fertilized normally. These fertilized eggs cleaved into good quality four-cell embryos which were frozen as the patient had to complete her therapy.[6] To the best of our knowledge, this is the first report of its kind for ovarian cancer. A similar approach has been applied for endometrial cancer.[5] This approach may not be applicable for patients without partners or for prepubertal patients. The age of the patient should be considered as the follicle reserve declines with age. Follicles undergo accelerated atresia in women after the age of 36[34,35] with early-growing and nongrowing follicles disappearing rapidly in ageing women.[35] Hence, ovarian tissue cryopreservation in aged patients may not be fruitful as their ovary may have very few follicles for cryopreservation.

## How to obtain ovarian tissue?

Medium to transport tissue to the lab

It is well known that ovarian cortex has hundreds of primordial and growing follicles. Therefore, only ovarian cortex is required for ovarian tissue cryopreservation. Ovarian cortex may be obtained using different surgical techniques as described by various reports mentioned above. The ovarian cortex should be delivered in a suitable medium to the cryopreservation lab. Leibovitz L-15 (Life Technologies) medium has been used by various authors according to the method described by Gosden *et al*.[11] to transport ovarian tissue after surgery to the laboratory. Some authors transported ovarian tissue in standard IVF medium on ice from a satellite center to the specialist center.[36] The transport lasted for 3–4 h including a flight journey. Specially formulated medium has also been used in experimental approaches.[37] HEPES-buffered human tubal fluid supplemented with human serum albumin was used to transport ovarian tissue to the laboratory and the tissue was incubated in HTF with albumin in a CO_2_ incubator until slicing of the tissue was completed.[38] However, the same author has used HEPES-buffered Ham's F-10 later[39] to transport the tissue to the laboratory. Authors using Leibovitz L-15 kept the tissue and medium at 4°C whereas Gook and her team[38] kept the tissue at 37°C.

## Thickness of cortical slices

Removal of stromal tissue before slicing the cortex is considered to be important to reduce the thickness of the tissue to be frozen. Presence of stromal tissue may impair the permeation of the cryoprotectant into the ovarian cortex, thereby, resulting in poor or reduced survival rates of the follicles.[38–40] The thickness of the slices is kept optimal to facilitate equilibration of the cryoprotectant. Although most of the published reports kept the thickness of the slices at 1 mm, the surface area varied from 2 × 2 mm to 5 × 5 mm.[22,28,38–41] The ovarian cortex may be cut into bigger pieces for easier transportation to the specialist laboratory where final trimming could be done.[36]

## Cryoprotectant and dehydration time

Cryoprotectant and dehydration time are major factors in the cryopreservation of any tissue. DMSO, propanediol (PROH), ethylene glycol (EG) and glycerol have been used by various authors as cryoprotectants to cryopreserve ovarian tissue. Although PROH is the popular cryoprotectant to freeze early cleavage embryos, DMSO also appears to be widely used in ovarian tissue cryopreservation. Survival of primordial follicles was the poorest after freezing and thawing with glycerol as the cryoprotectant compared to DMSO, PROH and EG.[15] EG has given the best survival rate of follicles in the cryopreserved ovarian tissue.[15] DMSO and EG permeate tissue faster at 4°C than PROH and glycerol, but PROH reached almost 100% equilibration at 37°C while the other three cryoprotectants reached 81% equilibration only after 30 min of incubation.[42] Therefore, the combination of temperature and the cryoprotectant must be taken care of while freezing ovarian cortex.

Initially, cryopreservation protocols for ovarian tissue did not have sugar in the cryoprotective medium.[14,15] Addition of sugar to the cryoprotecting medium as a nonpermeating cryoprotectant may prevent cryoinjury of the tissue[42] besides being an osmotic buffer against the osmotic stress caused by the permeating cryoprotectant.[43] The significance of sugar in the cryoprotective medium is evident from the normal histological appearance of cryopreserved tissue after thawing in most of the protocols with sucrose compared to those without sugar as shown in a comparative account.[44] Although Newton *et al*.[42] observed no significant improvement in the survival of frozen-thawed ovarian tissue with the addition of a low concentration of sugar to the medium, they believed that the inclusion of sugar is an added precaution against cryoinjuries. Appreciating the benefit of adding sugar, latter protocols also included sugar in the cryprotective medium.[28,36,38,40,41]

No standard dehydration time or duration of incubation of ovarian tissue in the cryoprotective medium has been found in the literature; the duration varies from 15 to 90 min.[14,15,38–40] Gook[38] has observed the highest proportion (91%) of intact oocytes in frozen-thawed ovarian tissue that has been dehydrated for 90 min with PROH and sucrose at 37°C compared to those dehydrated for 15, 30 and 60 min. However, vacuoles were present in the oocytes of ovarian tissue that had been dehydrated in a single step for 90 min. Reduction in the dehydration time to 15 min may prevent vacuole formation.[44] Generally, protocols using DMSO or EG dehydrate tissue for 30 min generally. The number of steps in dehydration (one *vs* two) does not seem to make much difference although Gook *et al*.[38] have shown that single-step dehydration in PROH with sucrose has yielded the highest proportion of intact primordial and primary follicles in the cryopreserved human ovary.

## Freezing program

Most of the studies have used the Planer programmable freezer to cryopreserve human ovarian tissue. Protocols using DMSO or EG start freezing at 0°C while those using PROH start near room temperature. Seeding temperature also varies among the studies from -6°C to -9°C although most of the studies seed at -8°C. The program is similar in most of the reports with a cooling rate of -2°C/min to the seeding temperature followed by -0.3°C/min to -30°C (or -50°C) and finally, -50°C/min to -140 to -150°C[14,15,36,38] followed by storage in liquid nitrogen.

## Methods of thawing

Thawing of frozen ovarian tissue also varies in the published reports. Newton *et al*.[42] applied simple thawing at room temperature or with rapid agitation in a water bath at 20°C for 1–2 min. The frozen tissue could be thawed rapidly in a 37°C water bath for quality control of the cryopreservation procedure.[36] Gook *et al*.[38] have also used a 37°C water bath to thaw rapidly for 2–3 min followed by a thawing protocol used for human oocytes. Radford *et al*.[39] have thawed the tissue at room temperature for 30 sec and in a 37°C water bath for 1 min followed by three 5-min rolling washes in a large quantity of Leibovitz medium. However, much simpler thawing was used in the report on the world's first livebirth from ovarian tissue cryopreservation and transplantation.[20] The frozen tissue was thawed at room temperature for 2 min followed by immersion in a 37°C water bath. A different thawing method was used by Demeestere *et al*.[22] in which tissues were thawed at room temperature for 2 min and in a 25°C water bath for 2 min followed by washing for 5 min each in progressively lower concentrations of DMSO. Apparently, all these methods worked well; a common feature in all these methods is the use of a water bath at 37°C.

## Role of vitrification in human ovarian tissue cryopreservation

The pregnancies and livebirths reported from human ovarian cryopreservation and transplantation resulted from slow freezing of ovarian tissue. Gook *et al*.[38] have documented that rapid freezing of ovarian tissue resulted in a lower proportion of intact oocytes and a higher proportion of vacuolated oocytes. Attempts to vitrify human ovarian tissue have not given acceptable results due to the increase in necrosis observed in vitrified human ovarian tissue.[45] Vitrification has been shown to cause extensive damage to the ovarian tissue not only in human tissue but also in bovine ovarian tissue.[46] However, a recent report on a novel technique of needle immersion vitrification yielded satisfactory survival of follicles in both human and murine ovarian tissue.[47] Vitrification of human ovarian tissue has to be improved for wider application or to replace slow freezing; until then, slow freezing remains the golden standard for ovarian tissue cryopreservation.

## Sites of transplantation

Orthotopic and heterotopic sites were studied for transplantation of frozen-thawed ovarian tissue. The existing menopausal ovary has been the popular site in orthotopic transplantation studies.[20,22,23,39] Although these studies confirm the menopausal status of the ovary, resumption of ovarian function postcancer therapy cannot be ruled out. Autologous heterotopic transplantation of ovarian tissue to a suprapubic site has also been reported by Oktay to result in spontaneous pregnancy and livebirth.[48] The patient in this study had been menopausal for 2.5 years during which, she did not get pregnant after having unprotected sexual intercourse. She did, however, get pregnant three months after the heterotopic transplantation. The author who raised suspicion on the source of the pregnancy and livebirth[21] reported by Donnez *et al*.,[20] noted with caution that the pregnancy could have occurred from spontaneous recovery of function in the existing menopausal ovary. However, recovery of ovarian function induced by transplanted tissue is very unlikely. Heterotopic transplantation into sites such as the forearm or lower abdomen may be beneficial if IVF treatment is attempted, as spontaneous conception from these sites is impossible.[18,19]

## Additional approaches with ovarian tissue cryopreservation

Collection of oocytes and ***in vitro*** maturation

An alternative means of fertility preservation is feasible in addition to ovarian tissue cryopreservation. Fresh ovarian cortex may be transplanted to monozygotic twins who are discordant for ovarian failure.[32,33] Immature oocytes may be collected from the visible follicles of the ovarian cortex prior to cryopreservation as antral follicles may be present. Such immature oocytes may be frozen after *in vitro* maturation.[28,49] On the other hand, if the patient has a partner, the *in vitro* matured oocytes could be fertilized by ICSI and the resulting embryos could be frozen.[5,6,40] This approach is more suitable for patients with endometrial or ovarian cancer in whom transplantation of ovarian tissue is avoided for fear of reintroducing cancerous cells.

## Cryopreservation of the whole ovary

Cryopreservation of the whole ovary has been attempted as an alternative to ovarian cortical slices as these slices suffer from loss of follicles mainly from ischemic damage and sometimes due to cryoinjury.[25,50] Transplanting the intact ovary may avoid ischemic damage and maintain viability and function of the tissue. Cryopreserving the whole organ is a challenge due to its size and the involvement of larger quantities of tissue and various biomolecules. High postthaw survival of follicles and well preserved stromal and vascular tissue has been shown in intact human ovary when cryopreserved with pedicle.[51,52] These papers demonstrated successful perfusion of cryoprotectant through the ovarian artery followed by slow cooling. Although these advances are promising, cryopreservation of the intact ovary has a long way to go before its application to patients for an effective treatment.

## Ethical concerns in ovarian tissue cryopreservation

The account given so far clearly shows that ovarian tissue cryopreservation is still experimental, although pregnancies from this technique have been reported. Therefore, the patient should be counseled thoroughly that this technique is purely experimental and that the risk of reintroducing cancerous cells, although very rare, is not ruled out. Parents should be given available information and future prospects on ovarian tissue cryopreservation if the patient is a minor. Using the tissue is entirely left to the option of minor patients when they enter adulthood, as starting a family is their choice. Getting informed consent after conveying the relevant medical information explaining the risks and uncertainties would help adult patients and parents of minor patients to avail the technique of ovarian tissue cryopreservation. The article by Van Den Broecke *et al*.[53] is recommended for more information on the ethical reflections on this technique.

Ovarian tissue cryopreservation is a promising mean of fertility preservation. Although spontaneous pregnancies from ovarian tissue cryopreservation and transplantation have been reported, the technique is still considered to be experimental. The source of pregnancy after ovarian tissue transplantation from ovarian transplants or the recovered existing menopausal ovary is still unclear, although evidence is in favor of transplants. Cryopreservation of the intact ovary and concerns about ethical issues related to this technique have to be explored further.
